# Psychometric properties of the Chilean version of the quality of life questionnaire for multiple myeloma

**DOI:** 10.1590/0034-7167-2023-0100

**Published:** 2024-05-03

**Authors:** Luz Alejandra Lorca, Cinara Sacomori, Camila Peña, Claudia Barrera, Melissa Salazar, Ivana Leão, Ximena Valladares, Christine Rojas

**Affiliations:** I Hospital del Salvador. Santiago, Chile; II Universidad del Desarrollo, Facultad de Medicina, Clínica Alemana. Santiago, Chile; III Hospital Gustavo Fricke. Viña del Mar, Chile; IV Universidad Católica del Maule. Talca, Chile

**Keywords:** Multiple Myeloma, Quality of Life, Validation Study, Psychometrics, Pain., Mieloma Múltiplo, Qualidade de Vida, Estudo de Validação, Psicometria, Dor., Mieloma Múltiple, Calidad de Vida, Estudios de Validación, Psicometría, Dolor.

## Abstract

**Objectives:**

To evaluate the internal consistency and construct validity of the QLQ-MY20 for assessing the quality of life in multiple myeloma survivors in Chile.

**Methods:**

This was a cross-sectional study conducted between March 2020 and December 2022. It involved 118 individuals from two public hospitals. The QLQ-C30 and QLQ-MY20 questionnaires were used. Internal consistency was assessed using Cronbach’s alpha(α), and construct validity was evaluated through hypothesis testing (Mann-Whitney and Spearman correlation).

**Results:**

The average age of participants was 67.2 years (SD=9.2). Internal consistency for the complete scale was α=0.779, for the “disease symptoms” dimension α=0.671, for the “side effects of treatments” dimension α=0.538, and for the “future perspective” dimension α=0.670. Four of the five construct validity hypotheses were confirmed: women, individuals with worse performance status, those with pain, and those with worse fatigue showed more symptoms.

**Conclusions:**

The Chilean version of the QLQ-MY20 demonstrates adequate internal consistency and construct validity.

## RESUMEN

**Objetivos:**

Evaluar consistencia interna y validez de constructo del QLQ-MY20 para valoración de calidad de vida en sobrevivientes de mieloma múltiple en Chile.

**Métodos:**

Estudio transversal, realizado entre marzo 2020 y diciembre 2022. Participaron 118 personas de dos hospitales públicos. Se utilizaron los cuestionarios QLQ-C30 y QLQ-MY20. Fueron evaluadas la consistencia interna con alfa de Cronbach (α) y validez de constructo mediante pruebas de hipótesis (Mann Whitney y correlación de Spearman).

**Resultados:**

El promedio de edad de los participantes era 67,2 (DE=9,2) años. Consistencia interna para escala completa (α=0,779), dimensión “síntomas de la enfermedad” (α=0,671), dimensión “efectos secundarios de los tratamientos” (α=0,538) y dimensión “perspectiva de futuro” (α=0,670). Se comprobaron cuatro de las cinco hipótesis de la validez de constructo: presentaron más síntomas las mujeres, personas con peor performance estatus, con dolor y con peor fatiga.

**Conclusiones:**

La versión chilena del QLQ-MY20 presenta adecuada consistencia interna y validez de constructo.

## RESUMO

**Objetivos:**

Avaliar consistência interna e validade de construto do QLQ-MY20 para avaliação da qualidade de vida em sobreviventes de mieloma múltiplo no Chile.

**Métodos:**

Estudo transversal, realizado entre março de 2020 e dezembro de 2022. Participaram 118 pessoas de dois hospitais públicos. Foram utilizados questionários QLQ-C30 e QLQ-MY20. A consistência interna foi avaliada com alfa de Cronbach(α) e a validade de construto através de testes de hipóteses (Mann Whitney e correlação de Spearman).

**Resultados:**

A idade média dos participantes era de 67,2 (DP=9,2) anos. Consistência interna para escala completa (α=0,779), dimensão “sintomas da doença” (α=0,671), dimensão “efeitos colaterais dos tratamentos” (α=0,538) e dimensão “perspectiva de futuro” (α=0,670). Quatro das cinco hipóteses de validade de construto foram confirmadas: as mulheres apresentaram mais sintomas, assim como pessoas com pior estado de desempenho, com dor e com maior fadiga.

**Conclusões:**

A versão chilena do QLQ-MY20 apresenta consistência interna adequada e validade de construto.

## INTRODUCTION

Multiple Myeloma (MM) is a neoplasm of plasma cells, accounting for 10% of hematological neoplasms^(1)^. It is primarily diagnosed in older adults, with the median age at diagnosis in Chile’s public system being 65 years. However, a third of diagnosed patients are under 60 years old^(2)^. In Chile, epidemiological data collected in the Second National Cancer Surveillance Report 2020 estimated the incidence of MM, by gender, at 4.3 cases per 100,000 inhabitants per year in men and 3.2 cases per 100,000 inhabitants per year in women, for the period from 2003-2010. This period saw 853 new cases reported annually and a prevalence of 2207 cases over five years^(3)^. Both in Chile and in Latin America^(4)^, the diagnosis is often made in more advanced stages of the disease, which tends to have higher morbidity and mortality compared to cohorts from developed countries^(5)^. Advances in screening and new therapeutic options, however, have improved survival rates, and health-related quality of life (HRQoL) has become an important therapeutic goal^(6)^.

Patients diagnosed with MM have shown significant improvements in overall survival, leading to a considerable number of older people living with the disease^(7)^. However, due to the effects of the disease itself and its treatments, patients experience deterioration in physical function, emotional and psychological well-being, social well-being, and work capability^(8)^. Additionally, they suffer from a significant symptom burden, such as fatigue and pain, which can significantly affect their quality of life, especially in older individuals^(9)^.

Survivors of MM experience substantial long-term quality of life issues and are second only to lung cancer patients in terms of the worst HRQoL across all cancer types^(10)^. It has also been shown that MM patients have the lowest HRQoL scores compared to patients with other hematological cancers^(11)^. Traditionally, studies in onco-hematology have considered objective clinical outcome measures, such as clinical response or survival. However, both patients and clinical researchers now argue that subjective outcome measures, such as HRQoL, should also be considered^(12)^.

Considering the relevant and specific problems related to MM, the European Organization for Research and Treatment of Cancer Quality of Life Group (EORTC)^(13)^ created and validated the specific module Quality of Life Questionnaire for Myeloma (QLQ-MY20)^(14)^. The QLQ-MY20 questionnaire has been validated in many countries^(9,12,15-16)^ and is relevant in the clinical management of MM patients, as it has the potential to improve treatment outcomes^(17)^. The questionnaire is available in a Spanish version translated for Chile, but its psychometric properties have yet to be evaluated. Validating the QLQ-MY20 questionnaire for the Chilean population will allow for the assessment of the perceived quality of life and health status by patients in relation to their disease. Furthermore, HRQoL research is currently gaining more importance as it is a relevant outcome in clinical studies, alongside survival, treatment efficacy, and patient adaptation to their disease.

## OBJECTIVE

To evaluate the internal consistency and construct validity of the QLQ-MY20 questionnaire for assessing Health-Related Quality of Life (HRQoL) in survivors of multiple myeloma in Chile.

## METHODS

### Ethical Considerations

This study was conducted in accordance with national and international ethical guidelines and was approved by the Scientific Ethics Committee of the Metropolitan East Health Service on 3^rd^ of March 2020, with their opinion attached to this submission. All participants gave their informed consent in writing.

### Study Design

This was an observational, cross-sectional validation study. The study was conducted between March 2020 and December 2022 in two public hospitals in Chile located in Santiago (Hospital del Salvador) and Viña del Mar (Hospital Gustavo Fricke). Reporting guidelines for validation studies from COSMIN (COnsensus-based Standards for the selection of health Measurement INstruments) were followed^(18)^.

### Population and Setting

The study population consisted of adult survivors of MM, who were patients at two Chilean public hospitals, regularly attending Hematology and Physical Medicine and Rehabilitation services, and were in different stages of the disease. A total of 118 individuals were enrolled. The sample was of a consecutive type. Recruitment was carried out by five health clinical professionals (one hematologist and four physiotherapists) who conducted face-to-face interviews to apply the scales.

Exclusion criteria included patients with another associated neoplasm, critically ill patients, those with cognitive impairment or illiteracy (insufficient understanding of Spanish), and individuals with any hearing or cognitive impairment that prevented them from responding to the questionnaires. For the evaluation of cognitive dimensions, the abbreviated mini-mental test was used^(19)^.

The sample size was estimated based on the primary objective of the study. Statistical literature suggests a minimum of 5 participants per questionnaire item^(20)^; thus, considering the questionnaire has 20 items, it was estimated that a minimum of 100 individuals should be evaluated. All adults who met the eligibility criteria were evaluated until the desired sample size was achieved.

### Study Protocol

For the execution of this study, the EORTC Quality of Life Group authorized the use of their questionnaires, providing the Spanish version. The EORTC QLQ-C30 includes five functional scales (physical, role, cognitive, emotional, and social); three symptom scales (fatigue, pain, nausea, and vomiting); and a global health scale. Additionally, it incorporates six individual items assessing symptoms that cancer patients may experience, such as dyspnea, loss of appetite, sleep disorders, constipation, and diarrhea^(13)^. Each item is rated on a scale from 1 to 4 (1=not at all, 2=a little, 3=quite a bit, 4=very much). For the final calculation, the scores of the 30 items are summed and divided by 30, yielding a quality of life score ranging from 0 (very poor) to 100 (excellent) for the functional dimensions. For the symptom dimension, a higher score corresponds to a higher level of symptom burden, with 100 interpreted as “maximum symptom burden” and 0 as “no symptoms”^(13)^. The EORTC QLQ-C30 has been validated for Chile in patients diagnosed with breast cancer^(21)^.

The EORTC QLQ-MY20 was developed as a supplement to the EORTC QLQ-C30 to assess the quality of life of patients with MM^(13)^. It consists of 20 questions grouped into four dimensions assessing “future perspective” (3 items), “disease symptoms” (6 items), “treatment side effects” (10 items), and a single item on “body image”^(14)^. Responses are scored on a four-point scale, ranging from “1=not at all” to “4=very much”. Scores are linearly transformed to a 0-100 scale. Higher scores in the body image and future perspective scales represent better outcomes, while higher scores in the symptoms and side effects scales indicate worse outcomes. For HRQoL assessment, the EORTC group recommends using both questionnaires. For calculating and estimating the scores of both instruments, a detailed manual provided by the EORTC group was used, available on the EORTC website^(22)^.

Information was collected from clinical records regarding sociodemographic variables (age, gender, education, marital status, institution) and clinical variables (cancer treatments received, nutritional status, smoking habits, alcohol consumption, and presence of vertebral fractures). Additionally, fatigue was assessed with the Brief Fatigue Inventory^(23)^, and performance status with the ECOG (Eastern Cooperative Oncology Group)^(24)^.

### Analysis of Results and Statistics

The data were tabulated in Excel and analyzed using SPSS software version 20. Descriptive statistical methods such as frequency, mean, median, and interquartile range were used. Internal consistency was measured using Cronbach’s alpha (α), where values of α>0.70 were considered to indicate high internal consistency, and values between 0.5 and 0.7 were considered as indicating moderate internal consistency^(25-26)^. The normality of the data was tested with the Kolmogorov-Smirnov test and a normal curve graph for observing symmetry and kurtosis.

For the analysis of construct validity, hypothesis tests were conducted using the Mann-Whitney U test to determine how well the QLQ-MY20 could discriminate between independent patient subgroups. Five hypotheses were proposed, expecting that the “disease symptoms” and “treatment side effects” dimensions would present higher scores in [1] women; [2] those with worse performance status; [3] those with pain; [4] those with the presence of fractures; and [5] those with moderate/severe fatigue. For the “body image” dimension, better scores were expected in men. For the “future perspective” dimension, better scores were expected in men and in people without pain, with mild fatigue, and without fractures.

In this study, performance status was categorized as good (ECOG: 0-1) and poor (ECOG: 2-3). For the fatigue variable, the cutoff point given by item 3 of the instrument was used, with the following categorization: individuals without fatigue or with mild fatigue (scores from 0 to 3) and individuals with moderate or severe fatigue (scores from 4 to 10).

Finally, to identify clinical differences and overlaps, correlation analyses were performed between the dimensions of the QLQ-MY20 and the QLQ-C30, using Pearson’s correlation test. A 95% confidence level was established for all analyses.

## RESULTS

### Sample Characterization

This study involved 118 patients (Table 1). The average age was 67.2 years (SD=9.2; Median=68; Minimum value=41; Maximum value=86). The majority were men (51.6%). Regarding treatments, most were treated with chemotherapy (n=116; 98.3%); 42 individuals (35.6%) received radiotherapy, and only two (1.7%) underwent hematopoietic stem cell transplantation. Additionally, 54 individuals had experienced vertebral fractures (45.8%). Furthermore, 37 individuals (31.3%) presented with a performance status (ECOG 2 and 3) and were unable to take care of their work, needing to be in bed at least 50% of the time. The most frequent comorbidities were isolated musculoskeletal disorders (24.6%) and those associated with other comorbidities. The “moderate” level of fatigue was the most prevalent, observed in 66 participants (55.9%), and the majority reported pain in some part of the body (88.9%).

**Table 1:** Characteristics of Patients with Multiple Myeloma from Santiago and Viña del Mar, Chile (N=118)

Variable	n (%)
Sex	
Female	57 (48.3)
Male	61 (51.7)
Educational Level	
Incomplete Primary Education	15 (12.7)
Complete Primary Education	16 (13.6)
Incomplete Secondary Education	13 (11.0)
Complete Secondary Education	27 (22.9)
Technical	30 (25.4)
Incomplete University Education	2 (1.7)
Complete University Education	15 (12.7)
Marital Status	
Married	73 (61.8)
Cohabiting	2 (1.7)
Divorced/Separated	9 (7.7)
Single	24 (20.3)
Widowed	10 (8.5)
Institution	
HDS - Santiago	78 (66.1)
HFG - Viña del mar	40 (33.9)
Cancer Treatments	
Chemotherapy	116 (98.3)
Radiotherapy	42 (35.6)
Hematopoietic Stem Cell Transplant	2 (1.7)
Nutritional Status	
Normal Weight	44 (37.3)
Overweight	53 (44.9)
Obesity	21 (17.8)
Presence of Vertebral Fractures	54 (45.8)
Presence of Pain	105 (89)
Smoker	5 (4.2)
Ex-Smoker	49 (41.5)
Occasional Alcohol Consumption	53 (44.9)
ECOG Performance Status	
0	8 (6.8)
1	73 (61.9)
2	32 (27.1)
3	5 (4.2)

*HDS: Hospital del Salvador; HGF: Hospital Gustavo Fricke; ECOG: Eastern Cooperative Oncology Group.*

### Quality of Life Characterization

Regarding the characterization of the dimensions of health-related quality of life (Table 2), it is possible to identify that the functional dimensions with the lowest scores were role functioning, social functioning, global health, and future perspective. Meanwhile, among the items related to cancer-related symptoms, those with the greatest impact on quality of life were insomnia, pain, fatigue, and financial difficulties. The symptoms of nausea and diarrhea showed a floor effect (16.7% and 33.3%, respectively).

**Table 2:** Characterization of the Quality of Life Dimensions in Patients with Multiple Myeloma from Santiago and Viña del Mar, Chile (N=118)

Dimensions	Md (IQR)	Internal Consistency (Cronbach's Alpha)
*EORTC QLQ-MY20*		0.779
Disease Symptoms	33.3 (22.2)	0.671
Treatment Side Effects	23.3 (16.7)	0.538
Body Image	100 (33.3)	#
Future Perspective	66.7 (33.3)	0.670
*EORTC QLQ-C30*		0.835
Physical Functioning	73.3 (33.3)	0.839
Role Functioning	66.7 (16.7)	0.795
Emotional Functioning	75 (25)	0.834
Cognitive Functioning	83.3 (33.3)	0.322
Social Functioning	66.7 (33.3)	0.515
Global Health	50 (25)	0.849
Fatigue	33.3 (22.2)	0.688
Nausea	0 (16.7)	0.464
Pain	33.3 (16.7)	0.686
Dyspnea	0 (33.3)	#
Insomnia	33.3 (33.3)	#
Loss of Appetite	0 (33.3)	#
Constipation	16.7 (33.3)	#
Diarrhea	0 (33.3)	#
Financial difficulties	33.3 (33.3)	#

*Md=Median; IQR=Interquartile Range; # It could not be calculated for these dimensions since they only have one item.*

Table 2 describes the specific aspects related to the Health-Related Quality of Life (HRQoL) of multiple myeloma (MY 20). It is noteworthy that, in terms of symptoms associated with MM, the dimension that showed the greatest impact was “disease symptoms” (Median=33.3%) compared to “treatment side effects” (Median=23.3%). As for the functional dimensions, “future perspective” (Median=66.7%) showed a greater impact than “body image” (Median=100%), with the latter dimension exhibiting a tendency towards a ceiling effect.

### Psychometric Properties of the QLQ-MY20

The internal consistency of both questionnaires and each dimension was moderate (Table 2). A high Cronbach’s alpha of 0.779 was obtained for the total QLQ-MY20 questionnaire.

**Figure 1:**
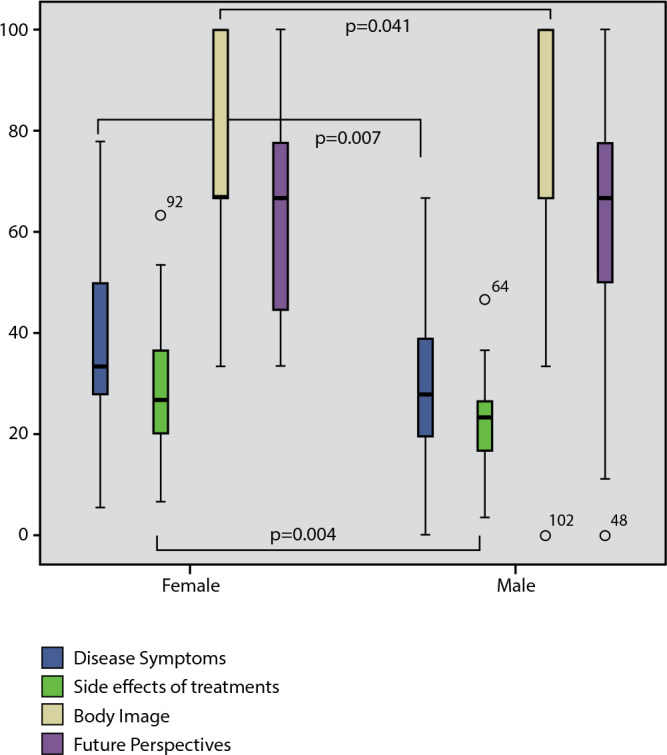
Comparison of EORTC QLQ-MY20 Scores Between Men and Women

Regarding the hypothesis tests for construct validity, four out of the five proposed hypotheses were confirmed. When comparing the QLQ-MY20 scores between men and women (Figure 1), it was identified that women had significantly lower “body image” scores (p=0.041) and higher scores in symptomatology (“disease symptoms” and “treatment side effects”; p=0.007 and p=0.041, respectively).

**Figure 2:**
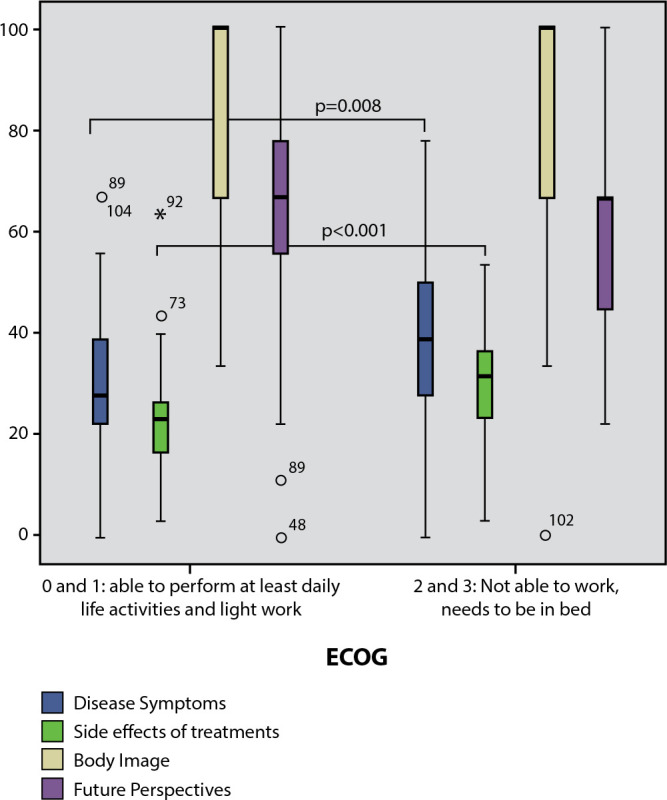
Comparison of EORTC QLQ-MY20 Scores Based on Performance Status (ECOG)

Patients with poor performance status (ECOG 2 and 3) significantly exhibited more “disease symptoms” and “treatment side effects” compared to patients with a good ECOG performance status (0 and 1) - See Figure 2.

Individuals with pain had higher scores for “disease symptoms” (p<0.001), “treatment side effects” (p=0.001), and lower “future perspective” (p<0.001) compared to those who did not report experiencing pain.

Patients with moderate/severe fatigue presented higher scores for “disease symptoms” (p=0.009), “treatment side effects” (p=0.035) compared to individuals without fatigue or with mild fatigue.

There was no statistically significant difference between individuals with and without vertebral fractures regarding the dimensions of the QLQ-MY20 (p>0.05).

Finally, most of the dimensions of the QLQ-MY20 were significantly correlated with the dimensions of the QLQ-C30 (Table 3). The strongest correlations were for the “disease symptoms” dimension of the QLQ-MY20 with the fatigue dimension (Spearman’s rho=0.594) and pain dimension (Spearman’s rho=0.663). Higher scores for “disease symptoms” and “treatment side effects” were associated with worse scores in physical functioning, role functioning, emotional, social, cognitive functioning, and global health (negative correlations, Spearman’s rho between -0.236 and -0.443).

**Table 3:** Correlations between the dimensions of the EORTC QLQ-MY20 and EORTC QLQ-C30 among patients with multiple myeloma from Santiago and Viña del Mar, Chile (N=118)

	Spearman’s rho			
	**Disease Symptoms**	**Treatment Side Effects**	**Body Image**	**Future Perspective**
Physical Functioning	-.415^^**^^	-.337^^**^^	.193^^*^^	.308^^**^^
Role Functioning	-.356^^**^^	-.309^^**^^	.167	.245^^**^^
Emotional Functioning	-.411^^**^^	-.382^^**^^	.280^^**^^	.325^^**^^
Cognitive Functioning	-.236^^*^^	-.420^^**^^	.226^^*^^	.114
Social Functioning	-.249^^**^^	-.247^^*^^	.258^^**^^	.198^^*^^
Global Health	-.443^^**^^	-.400^^**^^	.220^^*^^	.221^^*^^
Fatigue	.594^^**^^	.447^^**^^	-.239^^*^^	-.302^^**^^
Nausea	.084	.171	-.023	.022
Pain	.663^^**^^	.322^^**^^	-.272^^**^^	-.314^^**^^
Dyspnea	.339^^**^^	.278^^**^^	-.362^^**^^	-.302^^**^^
Insomnia	.298^^**^^	.276^^**^^	-.046	-.058
Loss of Appetite	.171	.172	-.139	-.161
Constipation	.096	.104	-.119	-.197^^*^^
Diarrhea	.079	.140	-.016	.053
Financial Difficulties	.236^^*^^	.174	-.144	-.138

** Significant for p<0.01;

* Significant for p<0.05.

## DISCUSSION

This study identified that the Chilean Spanish version of the QLQ-MY20 questionnaire for the assessment of Health-Related Quality of Life (HRQoL) demonstrated good results in terms of internal consistency and construct validity. Overall, the psychometric properties of the QLQ-MY20 were strong, similar to the data reported in other validation studies of the questionnaire^(9,12,15-16)^. Internal consistency showed adequate values for the complete questionnaire and moderate consistency for each dimension. The moderate internal consistency obtained for some scales may be a result of the small number of items in each domain and the heterogeneity of the studied population, rather than due to the low internal consistency of the items^(26)^.

Regarding construct validity, the results mostly met expectations, with 4 out of 5 hypothesis tests being confirmed. Low performance status was associated with worse HRQoL. Previous studies have also linked functional status to lower levels of functioning, higher symptom levels, and poorer quality of life^(11,27)^. In a German study, functional status, assessed with ECOG, was the strongest determinant of HRQoL^(28)^.

Concerning fatigue, it was confirmed that higher levels were associated with worse HRQoL. Fatigue, along with the reduction of physical functioning and breathing difficulties experienced by people with MM, has been known to contribute to poor HRQoL^(11,29)^. Additionally, fatigue has been linked to greater deterioration of daily activities, shorter disease-free progression, and overall survival^(29)^.

In this study, patients who reported experiencing pain had higher scores for the two symptom dimensions and lower scores for future perspective compared to those without pain. Bone pain is a significant symptom in MM^(30)^. Even when the disease is stabilized, 63% of patients report moderate or severe pain, and 80% report that pain affects their daily lives^(31)^. Pain can be present at different stages of the disease and may manifest as: bone pain induced by myeloma, peripheral neuropathic pain induced by chemotherapy, post-herpetic neuralgia induced by post-transplant immune depression, and pain in cancer survivors^(32)^.

Regarding gender, women experienced worse HRQoL than men in the dimensions of symptoms and body image. This finding, which has been reported previously, indicates that female gender, along with older age, predicts worse HRQoL in people with MM^(33)^. Additionally, a meta-analysis showed that, in general, men have higher levels of body appreciation compared to women, although the effect size was small^(34)^.

The only hypothesis not confirmed in this study was the influence of vertebral fractures on HRQoL, as there was no statistically significant difference between individuals with and without vertebral fractures. This result differs from the original validation study^(14)^ and the Iranian study^(15)^. A possible explanation is that the patients in this study were in better functional conditions, and those with fractures needing rehabilitation are promptly referred to a structured program existing in both participating institutions. Furthermore, patients can access technical aids and orthoses if required, which aims to enhance functioning and limit dependence.

In terms of the correlations between the QLQ-C30 and QLQ-MY20, the results support the construct validity. Scales that are conceptually correlated showed high correlations, while scales with less in common demonstrated lower correlations. This indicates that both questionnaires are robust and capable of providing reliable and precise descriptions of the HRQoL of patients with MM.

In this study, using the QLQ-C30 questionnaire, the dimensions most affected were role functioning, social functioning, and global health, which is similar to results obtained in other studies^(9,12,35)^. Regarding the symptom-related dimensions, fatigue, pain, insomnia, and financial problems were identified as having a greater impact on HRQoL, findings that are also described in other studies^(17,36)^. It has been reported that insomnia is common in most MM patients^(11)^, affecting caregivers and family members as well, and exacerbates other symptoms such as fatigue and pain^(37)^. Although the Chilean health system provides guaranteed access to MM treatment, some patients reported that due to the disease, loss of income and returning to work were challenging. Additionally, a significant percentage needed to cover extra expenses for transportation to hospital visits and for care.

Regarding the QLQ-MY20, participants in this study experienced a greater impact in the “disease symptoms” dimension, similar to the validation study of the Greek version^(9)^. In the “future perspective” dimension, a significant percentage were concerned about their future health and afraid of dying. The body image dimension, especially among men, did not appear to be greatly affected, aligning with what was reported in another study^(34)^.

### Study Limitations

One limitation was the lack of access to complete treatment duration records, which prevented the control of this variable. Additionally, it was not possible to perform a concurrent validity analysis due to the absence of a comparable instrument.

### Contributions to Nursing, Health, or Public Policy

This instrument, available in Spanish, will be useful for analyzing the clinical outcomes and HRQoL of patients with multiple myeloma who receive nursing care. Measuring HRQoL is crucial for guiding patient treatment in a comprehensive, systematic, and scientific manner.

## CONCLUSIONS

The Chilean version of the QLQ-MY20 questionnaire demonstrates adequate internal consistency and construct validity, making it a robust tool for assessing HRQoL in adult survivors of MM. Therefore, it is recommended for clinical application. The promotion and maintenance of quality of life should be integrated as objectives of clinical care and as criteria for systematic evaluation. An intervention model aimed at improving HRQoL to facilitate interdisciplinary rehabilitation in MM survivors is suggested. Additionally, future studies should investigate the instrument’s responsiveness in detecting changes in HRQoL related to different stages of the disease and following treatments.

## Data Availability

https://doi.org/10.48331/scielodata.KZR5M3
